# Neurotrophic factors in the porcine ovary: Their effects on follicular growth, oocyte maturation, and developmental competence

**DOI:** 10.3389/fvets.2022.931402

**Published:** 2022-08-10

**Authors:** Mirae Kim, Sang-Hwan Hyun

**Affiliations:** ^1^Laboratory of Veterinary Embryology and Biotechnology, Veterinary Medical Center and College of Veterinary Medicine, Chungbuk National University, Cheongju, South Korea; ^2^Institute of Stem Cell and Regenerative Medicine, Chungbuk National University, Cheongju, South Korea; ^3^Graduate School of Veterinary Biosecurity and Protection, Chungbuk National University, Cheongju, South Korea

**Keywords:** neurotrophic factors, pig, female reproduction, ovarian development, folliculogenesis

## Abstract

Pigs are cost-effective industrial animals because they produce a large number of offspring and have shorter rebreeding intervals compared with other animals, such as non-human primates. The reproductive physiology of pigs has been studied over the past several decades. However, there is not enough research on the effects of the neurotrophic factors on the ovarian physiology and development in pigs. As the ovary is a highly innervated organ, various neurotrophic factors during ovarian development can promote the growth of nerve fibers and improve the development of ovarian cells. Thus, investigating the role of neurotrophic factors on ovarian development, and the relationship between neurotrophic factors and porcine female reproduction is worth studying. In this review, we focused on the physiological roles of various neurotrophic factors in porcine ovaries and summarized the current status of the studies related to the relationship between neurotrophic factors and porcine ovarian development.

## Introduction

Neurotrophic factors, which are growth factors, play a crucial role in the regulation of neural survival, development, and function in both the central and peripheral nervous systems (1–3). They belong to one of the three main families: neurotrophins, glial cell line-derived neurotrophic factor (GDNF), or ciliary neurotrophic factor (CNTF) (4). Among these neurotrophic factors, the mammalian neurotrophin family consists of four members: nerve growth factor (NGF), brain-derived neurotrophic factor (BDNF), neurotrophin-3 (NT-3), and neurotrophin-4 (NT-4) (5). All neurotrophin families can bind to both the p75 receptor (p75^NTR^; pan-neurotrophin receptor) with low affinity and specific tropomyosin-related kinases (Trks; including TrkA, TrkB, and TrkC) with high affinity (6). GDNF, another neurotrophic factor, promotes the survival of dopaminergic neurons in the midbrain during embryonic development (7). CNTF can facilitate the survival and differentiation of ciliary ganglion neurons during embryonic development (8). These neurotrophic factors are not only closely related to the nervous system development (9–11), but they also affect the ovarian development (12, 13).

During ovarian development, sympathetic and sensory nerve fibers can reach most of the intraovarian tissues, including the follicles and interstitial tissues (14). As the ovary is a highly innervated organ, various neurotrophic factors during ovarian development can promote the growth of nerve fibers (15) and improve the development of ovarian cells, including theca, granulosa, cumulus, and oocytes (13, 16). The innervation of the ovaries is very important during folliculogenesis, especially in rodents, because the ovarian nerves first reach the gonads and then begin follicular assembly (17). Ovarian follicles are composed of oocytes and granulosa cell layers that surround them (18). Theca cells surround the basal lamina layers of the follicles (19) and are endocrine cells that exist only in the ovary, playing an important role in the follicular development. Ovarian folliculogenesis proceeds through the following steps: follicle assembly, primordial follicle activation, follicular growth, steroidogenesis, oocyte maturation, ovulation, and corpus luteum formation (20, 21). Since it is a highly regulated developmental process resulting in the growth, survival, and differentiation of oocytes and ovarian follicular cells (22), investigating the role of neurotrophic factors on follicular development and their association with mammalian female reproduction is essential.

Advances in reproductive biology have facilitated the development of assisted reproductive technologies, such as *in vitro* maturation (IVM), *in vitro* fertilization (IVF), and animal cloning by somatic cell nuclear transfer (SCNT). In particular, IVM is a creative technology that can supply high-quality oocytes *in vitro* and can be applied to biotechnology fields, such as conservation of endangered species, *in vitro* human-assisted reproduction, and transgenic animal production. Pigs are known as the most suitable animal for xenotransplantation and human disease modeling because their organ structure, size, and physiological characteristics are similar to those of humans (23). Thus, to successfully produce transgenic pigs, it is essential to establish both IVM and *in vitro* culture systems that can obtain high-quality oocytes and zygotes. Although assisted reproductive technologies for livestock are gradually developing, the efficiency of *in vitro* embryo production in pigs is still lower than that of natural breeding (24). In order to overcome such low efficiency of *in vitro* embryo production, numerous studies have been continuously performed to improve the developmental capacity of *in vitro* produced porcine embryos (25–27). Several factors are involved in the success of *in vitro* embryo production, one of which is the influence of intraovarian factors. In particular, there are various types of proteins, growth factors, and cytokines that exist in the porcine follicular fluid; however, their identity and functions are not well-known. Therefore, investigating the effects of various growth factors on porcine follicular development and *in vitro* embryo production systems could further accelerate the development of efficient transgenic pig production techniques, and the development of xenogeneic organs for human regenerative medicine and therapeutic applications (28). In this review, we noted the potential influence of neurotrophic factors in follicular growth, oocyte maturation, and embryonic developmental capacity in pigs.

Pigs also have the specific advantage of being cost-effective industrial animals because they produce a large number of offspring and have shorter rebreeding intervals than non-human primates (29). They are also a valuable model for understanding the process of follicular development since they are polytocous animals. The porcine estrous cycle is 18–24 days, of which the follicular phase comprises 5–7 days, and the luteal phase comprises 13–15 days (30). The porcine follicular dynamics compared with those of rodents have not yet been fully elucidated, and the follicular development, particularly during the estrous cycle, remains unclear (31). Nevertheless, many researchers have extensively studied the porcine estrous cycle and follicular development since they ovulate 15–30 oocytes at a time and give birth to multiple offspring (31–34). In addition, porcine ovaries are readily available from slaughterhouses, facilitating basic investigations of ovarian function (35). Their high offspring productivity helps to explore the knowledge about how to ovulate a large numbers of oocytes and to understand follicular development and female reproductive physiology.

The reproductive physiology of pigs has been studied over the past several decades (36); however, research on the effects of neurotrophic factors on the ovarian physiology and development in pigs is insufficient. Especially, the combination of these intra-ovarian neurotrophic factors play an important role in normal mammalian ovarian development. Therefore, the present review summarizes the physiological roles of various neurotrophic factors in porcine ovaries.

## The neurotrophin system in the porcine ovary

### NGF and its roles in ovarian and follicular development

NGF is the first and well-characterized member of the neurotrophin family (37). It binds to TrkA with high affinity. In general, NGF plays a vital role in the survival and maintenance of the sympathetic and sensory neurons (38). Numerous studies have demonstrated that NGF is also required for follicular growth following primordial follicle activation (39–41). NGF regulates the follicular assembly and development, and steroidogenesis in a cell culture model using human tissues (42, 43). In addition, NGF may participate in the pathogenesis of polycystic ovary syndrome (44, 45) and ovarian cancer (46) as shown by studies in rodents and humans. In particular, activation of the NGF/TrkA signaling pathway in human epithelial ovarian cancer cells can promote the metastasis of epithelial ovarian cancer by upregulating the expression of the vascular endothelial growth factor (46). Therefore, it is necessary to investigate the role of NGF in mammalian ovarian development.

Several studies have speculated that the interaction between NGF and its receptors can regulate porcine ovarian folliculogenesis, including the proliferation of follicular cells and stimulation of ovulation (47–49). Previous studies have investigated NGF and its receptors, TrkA and p75^NTR^, in porcine ovaries (48, 49). During porcine follicular development, the distribution of NGF and its receptor proteins in ovarian cells varies in each follicular stage (49). In the primordial and primary follicles, NGF and its receptors are not expressed in oocytes. However, with gradual development from primary follicles to secondary and tertiary follicles, this pattern changes, and both TrkA and its receptors are expressed in oocytes and follicular cells. Unlike in oocytes, NGF and its receptor proteins have been detected in follicular cells, which are present during all follicle maturation stages. NGF and TrkA proteins are the most highly expressed in the thecal and granulosa cells of large follicles among tertiary follicles (also known as antral follicles), which are classified as small, medium, and large. In contrast, the p75^NTR^ protein is highly expressed in all the tertiary follicular cells (48, 49). Interestingly, NGF and its receptors are also expressed in the steroidogenic cells of the corpus luteum (49).

Another study reported the effect of NGF supplementation during IVM on the porcine oocytes (47), showing that treatment with 1 ng/mL NGF for 30 hours during IVM significantly increased the rate of nuclear maturation. Therefore, the oocytes that matured in the presence or absence of 1 ng/mL NGF were used to investigate the subsequent embryonic development following IVF; however, NGF did not contribute to the improvement in embryo developmental competence. The mechanism underlying the effects of NGF on oocyte maturation and subsequent embryonic development has not been identified. Thus, previous studies suggested that the interaction of NGF and its receptors is involved in porcine follicular growth, steroid hormone production, ovulation, and corpus luteum formation, which are similar to their roles in rodents and humans (6, 40).

### BDNF and NT-4 and –their roles in ovarian and follicular development

BDNF promotes the survival and differentiation of several neurons, such as sensory and dopaminergic neurons (50). It was originally purified from pig brains in 1982 (51). BDNF is expressed not only in neurons, but also in all the other cells in the body, including immune cells (such as T cells, B cells, and monocytes), endothelial cells, and ovarian cells (52, 53). BDNF binds to TrkB with high affinity, and its interaction can activate angiogenesis, apoptosis, and cell survival pathways (54, 55). It also contributes to follicular development, and oocyte maturation in the mammalian ovary (53, 56–59). Paredes et al. (59) demonstrated that BDNF/NT-4 signaling pathway *via* TrkB is essential for oocyte survival during preantral follicular development by confirming oocyte death and loss of follicular organization in the ovaries of TrkB-null mice. Previous studies demonstrated that BDNF enhances *in vitro* oocyte maturation in humans (60), mice (61, 62), and cattle (63, 64). Furthermore, preimplantation embryonic development studies in cattle (57, 65) and mice (53, 66) have revealed that BDNF is present in the trophectoderm cells of the blastocyst, and BDNF supports early embryonic development.

Several studies have demonstrated BDNF to play a crucial role in supporting oocyte maturation (60, 62, 63, 67). In a human study, BDNF accelerated both nuclear and cytoplasmic maturation and enhanced the developmental potential following subsequent embryonic development (68). BDNF has been identified in human follicular fluid (69), and it is expressed in the granulosa cells and oocytes of the pre-antral follicles (70), and also granulosa cells and cumulus cells of the antral follicles (61, 71). In a bovine study, both BDNF and p75 mRNAs and proteins were present in the cumulus cells and oocytes, but the mRNAs of the full-length and truncated TrkB isoforms were detected only in the cumulus cells (65). While BDNF may have a positive effect on early ovarian follicular development, only a few studies have been reported in pigs.

BDNF promotes nuclear and cytoplasmic maturation of porcine oocytes and improves the developmental competence of embryos following IVF and SCNT (72). According to an aforementioned study (72), *BDNF*, truncated isoforms of *TrkB* (*trTrkB*), and *p75*^*NTR*^ mRNA transcripts were detected in both the porcine follicular cells and metaphase I oocytes. However, full-length *TrkB* mRNA transcripts were not observed in the metaphase I oocytes, but only in the follicular cells (granulosa and cumulus cells). The study also reported that treatment with a combination of BDNF and epidermal growth factor (EGF) during IVM improved the rate of blastocyst formation in embryos following IVF and SCNT, suggesting a synergistic effect between BDNF (30 ng/mL) and EGF (10 ng/mL) during subsequent embryonic development.

Another study also demonstrated that both mRNA transcripts and proteins of BDNF and TrkB were expressed in porcine granulosa cells and oocytes (73). In particular, Li et al. (73) reported that micro-RNA-205 (miRNA-205) was found to mediate BDNF-treated IVM oocytes through the regulation of *PTX3* (cumulus expansion-related gene), its putative target gene. Their study demonstrated that 50 ng/mL BDNF treatment during porcine IVM significantly (*p* < 0.05) improved nuclear maturation and significantly (*p* < 0.05) increased *PTX3* gene expression in cumulus-oocyte complexes (COCs). In addition, it was demonstrated that BDNF upregulates *PTX3* in COCs by inhibiting the expression of miRNA-205 in the granulosa cells, and that the upregulated *PTX3* level enhances the cumulus cell expansion. Thus, previous studies demonstrated that the BDNF/TrkB signaling pathway is required to enhance the nuclear and cytoplasmic maturation of oocytes and improve the developmental potency of embryos in pigs (72, 73).

NT-4 (also referred to as NT-4/5 or NT-5) was originally identified in *Xenopus* oocytes (74). Both BDNF and NT-4 bind to the same specific receptor, TrkB. NT-4 is an essential factor for follicular assembly in humans and rodents (15, 70, 75–78). In human studies, both the NT-4 mRNA and protein were expressed in oocytes and pre-granulosa cells in the pre-antral follicles (77), and NT-4 supplementation enhanced follicular assembly *in vitro* (78). A recent study demonstrated that 100 ng/mL of NT-4 promotes the *in vitro* growth of pre-antral follicles in mice (79). NT-4 (10 ng/mL) also promotes meiotic progression and first polar extrusion in mouse oocytes (80).

Unlike BDNF, only one study has reported the functional role of NT-4 in porcine ovaries. According to Kim et al. (81), the mRNA transcripts of *NT-4*, full-length *TrkB, trTrkB*, and *p75*^*NTR*^ were detected in the granulosa cells, cumulus cells, and immature and mature oocytes in porcine antral follicles. At the protein level, NT-4 was mainly detected in the theca and granulosa cells, whereas p75^NTR^ was generally expressed in all the follicular cells. The total TrkB protein was mainly expressed in the theca, granulosa, and oocytes, whereas the phospho-TrkB protein was predominantly expressed in the granulosa, cumulus, and oocytes. Therefore, NT-4-related signaling pathways may act on the growth of antral follicles by confirming the presence of NT-4 and receptors in porcine antral follicular cells. In addition, the addition of 10 ng/mL NT-4 and 10 ng/mL EGF to the IVM medium affects the EGF receptor signaling pathway to enhance porcine nuclear and cytoplasmic maturation of oocytes and the developmental potential of parthenogenesis-derived embryos. This finding suggests that NT-4 plays an important role in follicular development and oocyte maturation in porcine ovaries.

### NT-3 and its roles in ovarian and follicular development

NT-3 is known as the third neurotrophic factor of the neurotrophin family (82, 83). It binds strongly to a specific receptor, TrkC, but can also interact with TrkA and TrkB with very low affinity (84). Several studies over the past decades have shown that NT-3 does not only contribute to the survival, differentiation, and growth of existing neurons but also it facilitates the growth and differentiation of new neurons and synapses (85, 86). NT-3 and TrkC interactions may enhance the survival of sympathetic neurons (87); however, only few studies have reported the effects of these interactions on ovarian development.

In a rat study, NT-3 protein was detected in the granulosa cells and oocytes of primordial and primary follicles in neonatal ovaries (postnatal 4-day-old rat ovaries), whereas in adult ovaries, it was detected in all types of ovarian cells derived from larger developing follicles (88). In both neonatal and adult rat ovaries, the TrkC protein was expressed in the theca cells of pre-antral and antral follicles, as well as in the oocytes of all the developing follicles (88). This study demonstrated that NT-3 can promote early follicular development, which involves the transition from primordial to primary follicles, in rats. In contrast with a study in rats, both NT-3 and TrkC proteins have been identified in the oocytes and granulosa cells of human pre-antral ovaries from fetuses, girls, and women (89). According to Seifer et al. (69) the NT-3 protein is localized in the theca cells and oocytes of human antral follicles. The TrkC protein has been detected in oocytes (71) and granulosa cells (71, 90) of human antral follicles. In hamsters, when ovaries were cultured with 100 ng/mL NT-3 *in vitro*, estradiol secretion was stimulated (91). In cows, the proliferation of theca cells was promoted when 100 ng/mL NT-3 was added to the cell culture medium (92). These data suggest that NT-3 may be involved in steroidogenesis in antral follicles (91, 92). However, the correlation between NT-3 and ovarian development in pigs has not yet been elucidated.

## The GDNF system in the porcine ovary

### GDNF and its roles in ovarian and follicular development

GDNF was first discovered in 1993 as a growth factor involved in the survival of dopaminergic neurons in the embryonic midbrain (7). The GDNF family is composed of four members: GDNF, neurturin, artemin, and persephin (93). Among these GDNF families, GDNF is a well-known growth factor. GDNF has two isoforms (a full-length isoform and a short isoform), and it interacts with two receptors: the GDNF family receptor-α1 (GFR-α1), and the tyrosine kinase receptor for rearrangement during transfection (RET) (94). GDNF first binds to the GFR-α1 receptor, and the GDNF-GFR-α1 complex interacts with RET. Numerous studies have shown that GDNF has positive effects such as promoting oocyte maturation (61, 95–99) and enhancing embryonic development (95–97, 100–102).

GDNF has also been detected in porcine follicular fluid (95). Treatment with GDNF alone or in combination with other growth factors enhances the porcine oocyte maturation and developmental potential *in vitro* (95, 98, 99, 101). Linher et al. (95), for the first time in pigs, reported that GDNF enhances the cytoplasmic maturation and developmental potential of porcine oocytes. According to an aforementioned study (95), mRNA transcripts of *GDNF, GFR-*α*1*, and *RET* were detected in the cumulus cells and oocytes in the porcine antral follicles. Both GDNF and GFR-α1 proteins were also expressed in the cumulus cells and oocytes in the antral follicles. In particular, they also confirmed the presence of GDNF protein in the porcine follicular fluid extracted from each small (1–3 mm diameter) and medium (4–6 mm diameter) follicles *via* western blotting, and confirmed that there was no significant difference in the expression level. Their study demonstrated that GDNF is involved in porcine follicular development, and oocyte maturation by confirming the presence of GDNF in the cumulus cells, oocytes and follicular fluid.

Toms et al. (99) provided the initial information on global transcriptomic changes in porcine oocytes following antral follicular growth from small (1–3 mm diameter) to medium (4–6 mm diameter) when GDNF (50 ng/mL) was added during IVM. These authors suggested that GDNF promoted the developmental capacity of porcine oocytes as the antral follicles grew. Other studies have reported the synergistic effects of GDNF and EGF during IVM (98, 101). The results of the aforementioned two studies (98, 101) suggest that supplementation of the IVM medium with a combination of optimal concentrations of GDNF (50 ng/mL) and EGF (50 ng/mL) improves nuclear and cytoplasmic maturation of porcine oocytes and subsequent developmental competence following IVF. Based on several previous studies, GDNF improves follicular growth and oocyte maturation, as well as the developmental competence of immature oocytes in pigs.

## The CNTF system in the porcine ovary

### CNTF and its roles in ovarian and follicular development

The neurotrophic factor, CNTF, is a member of the interleukin-6 (IL-6) type cytokines, including IL-6, leukemia inhibitory factor, and oncostatin M (103). It was originally discovered in chick ciliary ganglia (104). However, unlike other neurotrophic factors (NGF, BDNF, NT-3, NT-4, and GDNF), CNTF is primarily expressed only in glial cells and is essentially different from classical target-derived neurotrophic factors (105–107). In particular, CNTF is known as a pleiotropic and neuroprotective factor secreted in the brains of patients with neurodegenerative diseases, such as Huntington's disease (108, 109); however, its effect on normal embryonic development is still uncertain. Only one study has reported on the relationship between IL-6 type cytokines, such as CNTF and human female germ cell development (110); no significant change in the levels of expression of leukemia inhibitory factor and CNTF was observed in human fetal ovaries during the gestational stage. Nevertheless, the expression levels of glycoprotein-130 and leukemia inhibitory factor receptors, which are receptors commonly shared by IL-6 type cytokines, significantly increased during the gestational stage, indicating that the IL-6 type cytokines play an important role in human fetal ovarian development. However, due to the pleiotropic characteristics of CNTF, its role in mammalian reproductive physiology and neurology remains unclear.

## Conclusion and future aspects

**Figure 1:**
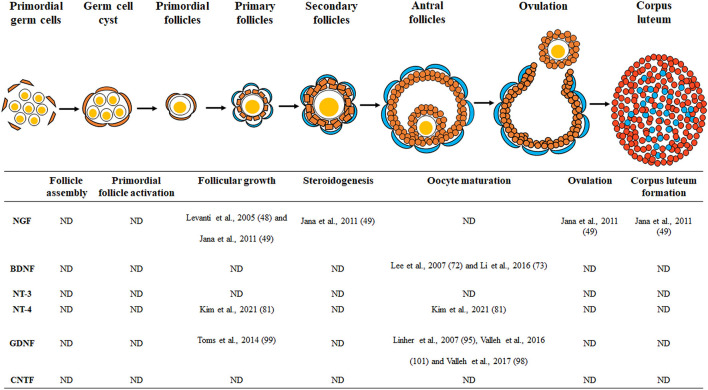
Schematic drawing of the neurotrophic factors involved in porcine ovarian and follicular development. Neurotrophic factors regulate porcine ovarian folliculogenesis, which involves follicle assembly, primordial follicle activation, follicular growth, steroidogenesis, oocyte maturation, ovulation, and corpus luteum formation. ND, no data.

During mammalian embryogenesis, various neurotrophic factors are associated with the differentiation of cells into specific tissues and organs. These neurotrophic factors and their receptors are known to affect ovarian development because they are present in the mammalian ovary. As shown in Figure 1 and Table 1, the role of neurotrophic factors in the porcine ovary is implicated in different events of early reproduction. However, there are still many unresolved questions about the physiological roles of neurotrophic factors in the porcine female reproductive system. Most of the existing studies have focused and analyzed on follicular growth, oocyte maturation, and early embryonic development; hence, the effects of intraovarian neurotrophic factors on follicular assembly, primordial follicle activation, hormone secretion, ovulation, and luteinization have not yet been studied. In addition, excess or deficiency of neurotrophic factors in the mammalian ovary in the development of reproductive disorders, such as polycystic ovary syndrome or infertility, is another matter to be discussed and should be further explained by future studies.

**Table 1:** The identification of various neurotrophic factors and their receptors in the porcine ovary.

**Ligands or receptors**	**Detection methods**	**Localization**	**Expression**	**References**
NGF	IF (49), WB (49)	Primordial follicles		(49)
		- Oocytes	–	
		Primary follicles		
		- Oocytes	–	
		- Follicular cells	+	
		Secondary follicles		
		- Oocytes	+	
		- Follicular cells	+	
		Tertiary follicles		
		- Oocytes	+	
		- Granulosa cells*	+	
		- Theca cells	+	
		Corpus luteus	+	(49)
		Follicular fluid	ND	–
TrkA	IHC (48), IF (49), WB (49)	Primordial follicles		(48, 49)
		- Oocytes	–	
		Primary follicles		
		- Oocytes	–	
		- Follicular cells	+	
		Secondary follicles		
		- Oocytes	+	
		- Follicular cells	+	
		Tertiary follicles		
		- Oocytes	+	
		- Granulosa cells*	+	
		- Theca cells	+	
		Corpus luteus	+	
p75^NTR^	IHC (48, 81), IF (49, 72), WB (49), RT-PCR (72, 81)	Primordial follicles		(49)
		- Oocytes	–	
		Primary follicles		(48, 49)
		- Oocytes	–	
		- Follicular cells	+	
		Secondary follicles		(48, 49)
		- Oocytes	+	
		- Follicular cells	+	
		Tertiary follicles		(48, 49, 81); excluding theca cells (72)
		- Oocytes	+	
		- Granulosa cells*	+	
		- Theca cells	+	
		Corpus luteus	+	(49)
BDNF	RT-PCR (72), IF (72)	Primordial follicles		
		- Oocytes	ND	–
		Primary follicles		–
		- Oocytes	ND	
		- Follicular cells	ND	
		Secondary follicles		–
		- Oocytes	ND	
		- Follicular cells	ND	
		Tertiary follicles		(72)
		- Oocytes	+	
		- Granulosa cells*	+	
		- Theca cells	ND	
		Corpus luteus	ND	–
		Follicular fluid	ND	–
NT-4	RT-PCR (81), IHC (81)	Primordial follicles		–
		- Oocytes	ND	
		Primary follicles		–
		- Oocytes	ND	
		- Follicular cells	ND	
		Secondary follicles		–
		- Oocytes	ND	
		- Follicular cells	ND	
		Tertiary follicles		(81)
		- Oocytes	+	
		- Granulosa cells*	+	
		- Theca cells	+	
		Corpus luteus	ND	–
		Follicular fluid	ND	–
TrkB	RT-PCR (72, 81), IF (72, 81), IHC (81)	Primordial follicles		–
		- Oocytes	ND	
		Primary follicles		–
		- Oocytes	ND	
		- Follicular cells	ND	
		Secondary follicles		–
		- Oocytes	ND	
		- Follicular cells	ND	
		Tertiary follicles		excluding theca cells (72); (81)
		- Oocytes	+	
		- Granulosa cells*	+	
		- Theca cells	+	
		Corpus luteus	ND	–
GDNF	qRT-PCR (95), IHC (95), WB (95)	Primordial follicles		–
		- Oocytes	ND	
		Primary follicles		–
		- Oocytes	ND	
		- Follicular cells	ND	
		Secondary follicles		–
		- Oocytes	ND	
		- Follicular cells	ND	
		Tertiary follicles		(95)
		- Oocytes	+	
		- Granulosa cells*	+	
		- Theca cells	ND	
		Corpus luteus	ND	–
		Follicular fluid	+	(95)
RET	qRT-PCR (95)	Primordial follicles		–
		- Oocytes	ND	
		Primary follicles		–
		- Oocytes	ND	
		- Follicular cells	ND	
		Secondary follicles		–
		- Oocytes	ND	
		- Follicular cells	ND	
		Tertiary follicles		(95)
		- Oocytes	+	
		- Granulosa cells*	+	
		- Theca cells	ND	
		Corpus luteus	ND	–
		Follicular fluid	ND	–
GFR-α1	qRT-PCR (95), WB (95)	Primordial follicles		–
		- Oocytes	ND	
		Primary follicles		–
		- Oocytes	ND	
		- Follicular cells	ND	
		Secondary follicles		–
		- Oocytes	ND	
		- Follicular cells	ND	
		Tertiary follicles		(95)
		- Oocytes	+	
		- Granulosa cells*	+	
		- Theca cells	ND	
		Corpus luteus	ND	–
		Follicular fluid	ND	–

*Granulosa cells collectively refer to mural granulosa and cumulus cells.

IF, immunofluorescence; WB, Western blot; IHC, immunohistochemistry; RT-PCR, reverse transcription polymerase chain reaction; qRT-PCR, quantitative RT-PCR; ND, no data.

## Author contributions

MK conceptualized the study, wrote, revised, and proofread the manuscript, and designed the table and figure. S-HH conceptualized the study, proofread the manuscript, and funded it. All authors have read and agreed to the published version of the manuscript.

## Funding

This work was supported, in part, by a grant from the National Research Foundation of Korea Grant funded by the Korean Government (2020R1A2C2008276), the Basic Research Lab Program (2022R1A4A1025557) through the National Research Foundation (NRF) of Korea, Korea Institute of Planning and Evaluation for Technology in Food, Agriculture, Forestry and Fisheries through the Agri-Bio Industry Technology Development Program (grant number: 318016-5), and Agriculture, Food and Rural Affairs Convergence Technologies Program for Educating Creative Global Leader (grant number: 320005-4), funded by the Ministry of Agriculture, Food and Rural Affairs, and the Global Research and Development Center Program through the National Research Foundation of Korea funded by the Ministry of Education, Science and Technology (2017K1A4A3014959), Republic of Korea.

## Conflict of interest

The authors declare that the research was conducted in the absence of any commercial or financial relationships that could be construed as a potential conflict of interest.

## Publisher's note

All claims expressed in this article are solely those of the authors and do not necessarily represent those of their affiliated organizations, or those of the publisher, the editors and the reviewers. Any product that may be evaluated in this article, or claim that may be made by its manufacturer, is not guaranteed or endorsed by the publisher.

## Abbreviations

BDNF: brain-derived neurotrophic factor
CNTF: ciliary neurotrophic factor
EGF: epidermal growth factor
GDNF: glial cell line-derived neurotrophic factor
IVM: *in vitro* maturation
NT: neurotrophin
IVF: *in vitro* fertilization
SCNT: somatic cell nuclear transfer
Trks: tropomyosin-related kinases.
